# Transient Supramolecular Hydrogels Formed by Aging‐Induced Seeded Self‐Assembly of Molecular Hydrogelators

**DOI:** 10.1002/advs.201902487

**Published:** 2020-02-05

**Authors:** Yiming Wang, Tomasz K. Piskorz, Matija Lovrak, Eduardo Mendes, Xuhong Guo, Rienk Eelkema, Jan H. van Esch

**Affiliations:** ^1^ State Key Laboratory of Chemical Engineering School of Chemical Engineering East China University of Science and Technology Meilong Road 130 200237 Shanghai China; ^2^ Department of Chemical Engineering Delft University of Technology van der Maasweg 9 2629 HZ Delft The Netherlands

**Keywords:** aging, hydrogels, pathway complexity, self‐assembly, supramolecular chemistry

## Abstract

Here, transient supramolecular hydrogels that are formed through simple aging‐induced seeded self‐assembly of molecular gelators are reported. In the involved molecular self‐assembly system, multicomponent gelators are formed from a mixture of precursor molecules and, typically, can spontaneously self‐assemble into thermodynamically more stable hydrogels through a multilevel self‐sorting process. In the present work, it is surprisingly found that one of the precursor molecules is capable of self‐assembling into nano‐sized aggregates upon a gentle aging treatment. Importantly, these tiny aggregates can serve as seeds to force the self‐assembly of gelators along a kinetically controlled pathway, leading to transient hydrogels that eventually spontaneously convert into thermodynamically more stable hydrogels over time. Such an aging‐induced seeded self‐assembly process is not only a new route toward synthetic out‐of‐equilibrium supramolecular systems, but also suggests the necessity of reporting the age of self‐assembling building block solutions in other self‐assembly systems.

## Graphical abstract

A simple aging‐induced seeded self‐assembly resulting in transient supramolecular hydrogels is reported. The obtained transient hydrogels can convert into the thermodynamically more stable state over time. The findings demonstrate a new route toward out‐of‐equilibrium self‐assembly system, and suggest the necessity of stating the ages of self‐assembling building blocks in some other supramolecular systems.

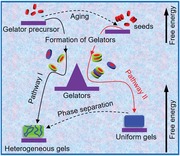

In this work, we present how transient supramolecular hydrogels can be formed by an aging‐induced seeded self‐assembly of molecular hydrogelators. Inspired by nature, synthetic supramolecular self‐assembly performed under out‐of‐equilibrium conditions has received extensive interest in recent years.^1^ The implementation of such a system in a manmade scenario would not only improve our understanding of the biological counterpart, but also result in supramolecular materials with numerous intriguing functions, such as adaptation,^2^ self‐replication,^3^ and associative learning.^4^ To date, most of the already developed examples of out‐of‐equilibrium self‐assembly are achieved through interfering in the self‐assembly kinetics of the subunits using, for instance, catalysts (enzymes),^5^ chemical fuels,^6^ and designed nucleation seeds.^7^ As a result, kinetically captured or transient supramolecular structures that function at out‐of‐equilibrium states are obtained.[qv: 7a,8] Here we show an aging‐induced seeded self‐assembly process of molecular gelators resulting in transient formation of supramolecular hydrogels.

Very recently, we have described a hydrazone‐based multicomponent hydrogelators system in which hydrazide (**H**) and aldehydes composed of neutral (**A**) and negatively charged (**A^–^**) species are coupled together to form neutral (**G**) and negatively charged hydrogelators (**G^–^**) through the in situ formation of hydrazone bonds (**Figure** **1**a).^9^ The resulting **G** and **G^–^** can self‐assemble into thermodynamically more stable heterogeneous hydrogels consisting of separated microdomains rich in neutral fibers (**F**) or negatively charged fibers (**F^–^**) through a multilevel self‐sorting process (Pathway I in Figure 1b).^9^ Our previous study has demonstrated that this multilevel self‐sorting mainly results from the different nucleation rates of **G** and **G^–^**.[qv: 7c]

**Figure 1:**
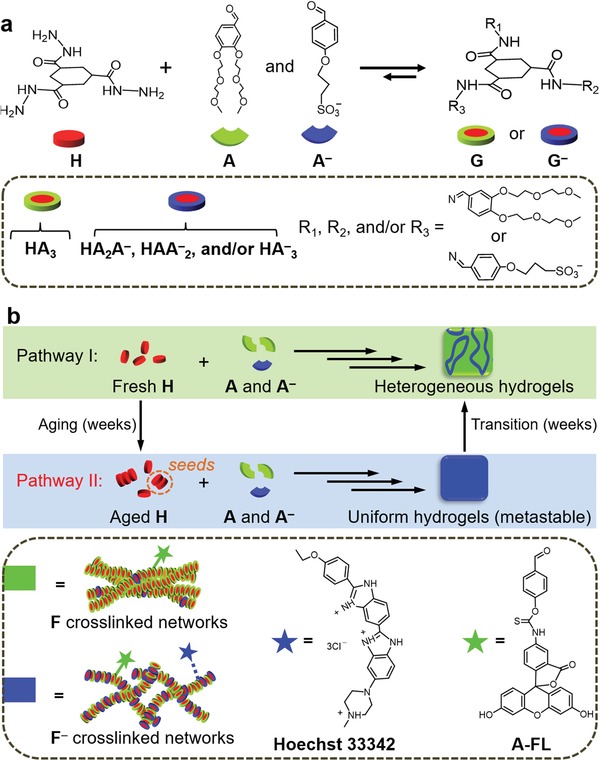
a) Scheme of the formation of **G** and **G^–^** from the building blocks of **H**, **A**, and **A^–^**; b) illustration of the self‐assembly of **G** and **G^–^** through different pathways leading to formation of different hydrogel products. Fluorescent probes of **A‐FL** and **Hoechst 33342** are used to label all the gel fibers and only **F^–^**, respectively.

In this work, we show that an aging treatment on the **H** solutions can lead to the formation of nano‐sized aggregates of **H**. These tiny aggregates of **H** can serve as seeds to interfere in the nucleation step of hydrogelator assembly, and direct their self‐assembly along a kinetically controlled pathway, resulting in the formation of kinetically favored hydrogels composed of **F^–^** (Pathway II in Figure 1b). These resulting kinetic hydrogels have a finite lifetime and can ultimately convert into thermodynamically more stable hydrogel states (Figure 1b). This work, on the one hand, shows a simple aging‐induced seeding approach toward transient hydrogel states yet without necessary addition of synthesized seeds, and on the other hand highlights the essential role of the age of self‐assembling building block solutions in a self‐assembly process.

All the hydrogel samples used in this work were prepared by mixing the aldehydes (**A** + **A^–^**) and **H** in phosphate buffer (0.1 m, pH 7.0). Excess aldehydes (six times higher than [**H]**) were used to ensure a complete conversion of **H** into tris‐hydrazone products.^10^ The age of **H** solutions used for aging‐induced seeded self‐assembly was 2 weeks or more, as at this time scale the aging effects leveled off and further aging did not cause additional acceleration effects on the self‐assembly. The self‐assembled structures were characterized using confocal laser scanning microscope (CLSM). To make the structures visible under CLSM, aldehyde functionalized fluorescein (**A‐FL**) (green channel) and cationic dye **Hoechst 33342** (blue channel) were used to label both types of self‐assembled structures, and the negatively charged structures, respectively (Figure 1b).

To make the hydrogels, phosphate buffered stock solutions of **H**, **A,** and **A^–^** were mixed together at a prescribed ratio and incubated at room temperature for occurrence of gelation. We have previously shown that turbid heterogeneous hydrogels with separated microdomains composed of **F** or **F^–^** can eventually be formed (Figure S1, Supporting Information).^9^ However, to our surprise, when a stock **H** solution that was stored for more than 2 weeks was used, the transparencies of the resulting hydrogels were increased with the amount of **A^–^** (Figure S2, Supporting Information). Such behavior is distinctly different from the previous turbid heterogeneous hydrogels prepared with relatively fresh **H** solutions. To gain further insight into this aging effect, we characterized the morphologies of the resulting hydrogel networks using CLSM. The hydrogel prepared with fresh **H** and pure **A** showed typical networks composed of large fibrous clusters, while the networks consisting of separated **F**‐rich and **F^–^**‐rich microdomains were formed with the addition of **A^–^** (**Figure** **2**a; and Figure S3, Supporting Information). All these observations match with a previous study.^9^ However, in case the aged **H** solution was used, distinctly different hydrogel network morphologies were obtained (Figure 2b). For the hydrogel formed with pure **A**, the size of the fibrous clusters was markedly decreased compared with the hydrogel prepared with fresh **H**. Moreover, with increasing the content of **A^–^**, the fibrous cluster content decreased. The hydrogel networks showed homogeneous fluorescence without any distinguishable structures when the content of added **A^–^** was >10 mol%. Cryogenic transmission electron microscopy (Cryo‐TEM) indicated that the homogenous hydrogel networks were mainly composed of thin fibers with a diameter of ≈5.8 nm (Figure S4, Supporting Information), which is comparable to a single hydrazone gelator fiber.^11^ These thin nano‐sized fibers scatter less light, thereby explaining the increased transparency of the hydrogels with increasing content of **A^–^**. Furthermore, **Hoechst 33342** staining tests revealed that the thin fibers in these homogenous hydrogel networks are **F^–^** (Figure S3, Supporting Information). Apparently, **F^–^** are not prone to form bundles due to the interfibrous electrostatic repulsions, thereby leading to the crosslinked homogeneous networks consisting of thin fibers. It is noteworthy that the dyes we used have no effects on the self‐assembled structures.^9^ These CLSM results clearly demonstrate that the use of aged **H** solutions prevents formation of heterogeneous hydrogels, instead leading to formation of **F^–^**‐based homogeneous hydrogels.

**Figure 2:**
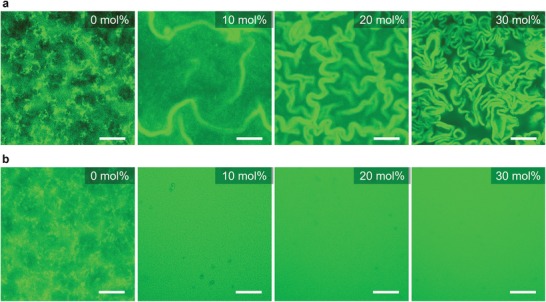
CLSM images of the hydrogel networks prepared with a) fresh and b) aged **H** solutions as a function of the amount of **A^–^**, scale bars = 40 µm. Samples: [**H**] = 20 × 10^−3^ m, [**A** + **A^–^**] = 120 × 10^−3^ m (different mol% **A^–^**), and [**A‐FL**] = 30 × 10^−6^ m.

The aging effects on the hydrogel network morphologies thereby lead us to investigate the corresponding material properties of the resulting hydrogels. To this end, oscillatory rheological measurements were performed to study the mechanical properties of the hydrogels. For the samples prepared with fresh **H** solutions, the gelation time (the time at which the storage modules *G′* surpass the loss modulus *G″*) was increased from ≈45 min to ≈2.65 h with increasing the content of added **A^–^** from 0 to 30 mol% (**Figure** **3**a); and the stiffness of the resulted hydrogels was first increased from ≈800 Pa (0 mol% **A^–^**) to ≈1.2 kPa (10 mol% **A^–^**) and then decreased to ≈60 Pa (30 mol% **A^–^**) (Figure 3b). These results are in line with a previous study.[qv: 7c] The use of aged **H** solution led to an increase in gelation time from ≈9 to ≈50 min with an increase in the content of **A^–^** from 0 to 30 mol% (Figure 3a), indicating faster gelation rates compared to the case using fresh **H**. Moreover, the stiffness of these hydrogels was between 2.6 and 5.6 kPa and showed only slight variations depending on the content of **A^–^** (Figure 3b). In all cases, these values are higher than for the hydrogels prepared with fresh **H** solutions. These rheological results clearly demonstrate that the use of aged **H** solution can have a marked effect on the gelation process and the resulting mechanical properties of the hydrogels.

**Figure 3:**
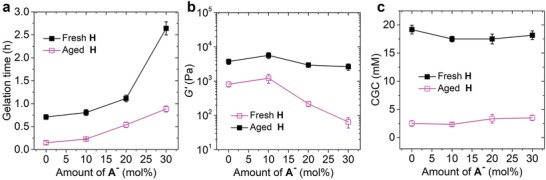
Effects of aged **H** solution on a) gelation time; b) hydrogel stiffness; and c) CGC. Samples in a,b): [**H**] = 20 × 10^−3^ m, [**A** + **A^–^**] = 120 × 10^−3^ m (different mol% **A^–^**). Error bars are calculated as s.d. of three measurements.

When measuring the critical gelation concentration (CGC), defined as the minimum concentration of **H** (see the Supporting Information), we noticed that hydrogels can only be formed with [**H**]_fresh_ > 17 × 10^−3^ m. However, the use of aged **H** solutions led to a stark decrease in CGC, varying from ≈2.5 to ≈3.5 × 10^−3^ m depending on the content of **A^–^** (Figure 3c). This remarkably lower CGC due to the use of aged **H** further confirms the accelerated gelation process.[qv: 8b]

The rate of gelator formation usually plays an important role in the gelation process.[qv: 8b,11,12] To investigate the mechanism of the observed accelerated gelation, we performed high performance liquid chromatography (HPLC) to analyze the kinetics of gelator formation. The results indicated that the addition of aged **H** solution did not change the hydrazone formation rates, nor the product compositions as compared with the case using fresh **H** solutions (Figure S5, Supporting Information). Moreover, ^1^H‐NMR test manifested that both the new and aged **H** showed the same resonances (Figure S6, Supporting Information), indicating that the aging treatment did not change the chemical structure of **H**. These HPLC results together with the ^1^H‐NMR test suggest that the observed accelerated gelation is ascribed to the interference of aged **H** in the self‐assembly process, rather than in the formation of the gelators. Such an accelerated self‐assembly process would lead to a fractal‐like growth of the fibers, which is responsible for the higher hydrogel stiffness.[qv: 8b,13]

It is necessary to note that the aging effects seem to be independent of the absorption of CO_2_, and in case we freeze‐dry the aged **H** solution and re‐dissolve the resultant **H** powders for the preparation of gel, heterogeneous gels are ultimately formed (Figure S8, Supporting Information). On the basis of these data, we propose an aging‐induced seeded self‐assembly mechanism. In this hypothesis, **H** molecules self‐assemble into nanoaggregates during aging. These nanoaggregates act as seeds to accelerate the self‐assembly of **G** and **G^–^** by interfering in the nucleation step. As a result, the seeded self‐assembly bypasses the multilevel self‐sorting process^9^ and leads to a co‐assembly of **G** and **G^–^**, ultimately resulting in homogeneous hydrogels exclusively composed of **F^–^**.[qv: 7c] Indeed, **H** molecules have the potential to form intramolecular interactions including hydrogen bonds between the hydrazide groups and hydrophobic interactions between the cyclohexyl cores, which drive the self‐assembly of **H** molecules during aging. This was confirmed by a simple molecular dynamic simulation (see Figure S7 and Movie S1 in the Supporting Information). To further test this hypothesis, we employed dynamic light scattering to check the aged **H** solution (see the Supporting Information). As compared to the control samples of blank phosphate buffer and fresh **H** solution, a scattering signal indicating structures with a 1.28 nm hydrodynamic diameter was detected in the aged **H** solution (Figure S9, Supporting Information), indicating that some nanostructures had indeed formed during aging. Furthermore, with addition of THF, a good solvent for **H**, into the aged **H** solution, the characteristic scattering peak at 1.28 nm disappeared (Figure S9, Supporting Information). Moreover, after evaporating THF away from the THF treated aged **H** solution, the resulting solutions led to formation of heterogeneous gels, indicating the vanishing of seeding effects (Figure S8, Supporting Information). These results further suggest the formation of **H**‐based nanoaggregates. However, we cannot distinguish these nanoaggregates in cryo‐TEM observations (Figure S10, Supporting Information), which could be ascribed to the small size and low contrast of the aggregates of **H**. It should be noted that the level off of the aging effects after 2 weeks can be ascribed to the self‐assembly of **H** molecules reaching at a dynamic equilibrium state where the rate of the self‐assembly is equal to that of the disassembly.

From the preceding results and discussions, we can conclude that the tiny aggregates of **H** formed during aging force the self‐assembly of **G** and **G^–^** along a kinetically favored pathway, where a co‐assembly of **G** and **G^–^** instead of the previously reported multilevel self‐sorting occurs, leading to the **F^–^** crosslinked homogeneous hydrogels.[qv: 7c] Interestingly, after an incubation of around 3 weeks, we found that the transparent hydrogel prepared with 30 mol% **A^–^** turned from transparent to turbid (**Figure** **4**). CLSM observations indicated that this turbid hydrogel showed identical network morphologies with the heterogeneous hydrogels. Moreover, in the blue channel we found that the crumpled structures showed higher intensity of blue fluorescence, while the fibrous networks in between showed relatively low blue intensity (Figure 4a). This result indicates that the crumpled structures are mainly composed of **F^–^**, while the networks in between are composed of **F**. Similar morphological transformations in the other hydrogel samples prepared with different mol% **A^–^** were observed as well (Figure S11, Supporting Information). It therefore demonstrates that these homogeneous hydrogels formed using aged **H** solutions are in metastable transient states and are capable of converting into thermodynamically more stable hydrogel states over time.

**Figure 4:**
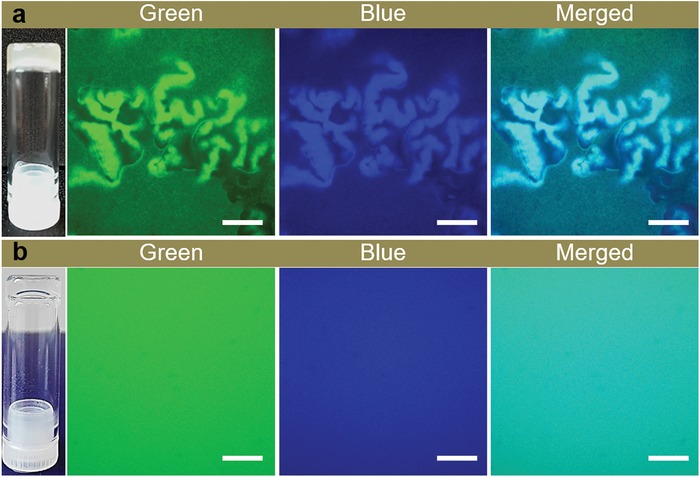
a,b) Photographs and CLSM images of the gel networks prepared with aged **H** solutions: a) after an incubation of 3 weeks; and b) the freshly formed hydrogel. Samples in a,b): [**H**] = 20 × 10^−3^ m, [**A** + **A^–^**] = 120 × 10^−3^ m (30 mol% of **A^–^**), [**A‐FL**] = 30 × 10^−6^ m, and [**Hoechst 33342**] = 20 × 10^−6^ m, scale bars = 40 µm.

In conclusion, we have presented the formation of out‐of‐equilibrium supramolecular hydrogels through an aging‐induced seeded self‐assembly process. One of the gelator precursor molecules self‐assembles into nano‐sized aggregates, which drive the self‐assembly of multicomponent gelators along a kinetically controlled pathway by interfering in the nucleation step, leading to kinetically favored hydrogel products. Interestingly, we have found that these hydrogels are in metastable transient states, and can convert into the thermodynamically more stable hydrogel states over time at room temperature. Our findings underline the potential importance of the ages of self‐assembling building blocks in a supramolecular system.^14^

## Conflict of Interest

The authors declare no conflict of interest.

## Supporting information

Supporting Information
