# Transient Supramolecular Hydrogels Formed by Aging‐Induced Seeded Self‐Assembly of Molecular Hydrogelators

**DOI:** 10.1002/advs.202003537

**Published:** 2021-08-04

**Authors:** Yiming Wang, Tomasz K. Piskorz, Matija Lovrak, Eduardo Mendes, Xuhong Guo, Rienk Eelkema, Jan H. van Esch

*Adv. Sci*. **2020**, *7*, 1902487

DOI: 10.1002/advs.201902487

In this originally published version of this article, the cryo‐TEM image in Supporting Information Figure S4 inadvertently features the wrong sample. The correct Figure S4 is presented below. The authors confirm that the error does not change any conclusions of the article, and apologize for this oversight.

**Figure S4:**
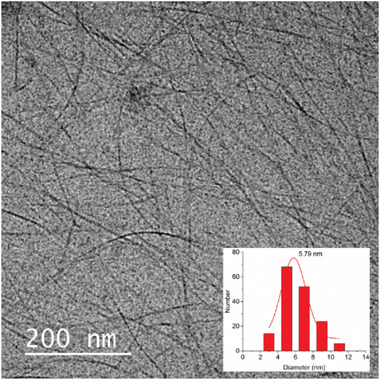
Cryo‐TEM image and statistical diameter (inset) of the hydrazone fibers.

