# Supplementary material for: Transient Supramolecular Hydrogels Formed by Aging‐Induced Seeded Self‐Assembly of Molecular Hydrogelators
Source: Adv Sci (Weinh). 2020 Feb 5;7(7):1902487. doi: 10.1002/advs.201902487 (PMC7140988; doi:10.1002/advs.201902487)
Supplement: Supplementary file 1 — Supporting Information [file ADVS-7-1902487-s001.pdf]

Copyright WILEY-VCH Verlag GmbH & Co. KGaA, 69469 Weinheim, Germany, 2019.

ADVANCED SCIENCE
Open Access

Supporting Information

for *Adv. Sci.,* DOI: 10.1002/advs.201902487

Transient Supramolecular Hydrogels Formed by Aging-Induced Seeded Self-Assembly of Molecular Hydrogelators

*Yiming Wang, Tomasz K. Piskorz, Matija Lovrak, Eduardo Mendes, Xuhong Guo, Rienk Eelkema, and Jan H.van Esch**

WILEY-VCH

Copyright WILEY-VCH Verlag GmbH & Co. KGaA, 69469 Weinheim, Germany, 2019.

Supporting Information

Transient supramolecular hydrogels formed by aging-induced seeded self-assembly of molecular hydrogelators

*Yiming Wang, Tomasz K. Piskorz, Matija Lovrak, Eduardo Mendes, Xuhong Guo, Rienk Eelkema, Jan H. van Esch**

Materials

All commercial chemicals were purchased from Sigma Aldrich. Compound **H**, **A**, **A¯**, and **A-FL** were synthesized according to methods described in our previous work.[1]

Experimental

Preparation of the stock solutions (H, A, and A¯) and gels

The stock solutions of **H**, **A**, and **A¯** were prepared by dissolving prescribed amounts of the corresponding compound in phosphate buffer (0.1 M, pH 7.0) which can well buffer the pH of the solutions at 7.0. The aging treatment of **H** solution was performed at room temperature. To rule out the contributions of CO_2 to the aging effects, **H** solution aged under argon atmosphere was prepared as a control. To prepare the gels, determined amounts of the stock solutions of **H** (aged or freshed), **A**, and **A¯** were mixed together and incubated at room temperature for the occurance of gelation.

Rheological test

Oscillatory rheological measurements were performed on a AR G2 TA rheometer equipped with a parallel-plate made of stainless steel (diameter is 40 mm) and a solvent trap to prevent the evaporation of water from the samples. All the measurements were performed in a strain controlled mode at 25 ^{o}C. The strain and the frequency for the time sweep measurements were set to 0.05% and 1.0 Hz, respectively. All the samples were prepared in 0.1 M phosphate buffer at pH 7.0. After mixing the stock solutions of the building blocks to the target concentration, 400 μL of the solution was transferred immediately onto the rheometer plate and the gap was set to be 290 μm for the measurements with a solvent trap.

Determination of the critical gelation concentration (CGC)

A series of samples include different concentration of **H** and (**A** + **A¯**) was prepared and kept at room temperature for one week to ensure a complete formation and self-assembly of gelators; different ratio of **A¯** was investigated as well, but the concentration of **H** was constantly maintained at six times higher than (**A** + **A¯**). After that, the gel formation was determined by vial inversion test. Typically, the resultant sample which can resist flow for at least 30 seconds was then regarded as gel in this study. The lowest concentration of **H** at which the sample was able to form a gel was defined as the corresponding CGC.

CLSM measurement

CLSM measurements were performed on a Zeiss LSM 710 confocal laser scanning microscope equipped with a Zeiss Axio Observer inverted microscope and 40x PlanFluor oil immersion objective lens (NA 1.3). Incident laser with a wavelength of 488 nm and 405 nm was used to excite the fluorescein and Hoechst probes, respectively. The pinhole was set to 1.0 airy unit during the measurements and the data were processed using ZEN 2009 software. To preserve the gel samples for longer time without dehydration, samples were prepared in sealed homemade PDMS chambers.

High performance liquid chromatography (HPLC)

The gelator composition of the gels was analyzed by high performance liquid chromatography (HPLC) using a gradient eluent flow of H_2O:(MeOH+0.4% triethylamine (TEA)) linearly varied from a ratio of 8:2 to 2:8, referring to the previous study.[1c] The

samples for the HPLC measurements were prepared by adding 80 μL gel sample into a mixture solution of 400 μL THF and 400 μL TEA saturated H_2O. The ratio of each gelator was calculated at isosbestic point (275 nm). The first increase followed by a decrease in the content of $\mathbf{HAA^{-}_{2}}$ and $\mathbf{HA^{-}_{3}}$ can be ascribed to the occurrence of self-assembly; $\mathbf{HA_3}$ and $\mathbf{HA_2A^{-}}$ are easier to self-assemble because of their less charges; the self-assembly of $\mathbf{HA_3}$ and $\mathbf{HA_2A^{-}}$ leads to the decrease of their content in water, which leads a shift of the reaction equilibrium to the formation of $\mathbf{HA_3}$ and $\mathbf{HA_2A^{-}}$. As a result, the $\mathbf{HAA^{-}_{2}}$ and $\mathbf{HA^{-}_{3}}$ go backward to the intermediate molecules which can convert to $\mathbf{HA_3}$ and $\mathbf{HA_2A^{-}}$ to supplement their decreased content in water.

Cryo-TEM

A Gatan model 626 cryo-stage in a JEOL JEM 1400 Plus electron microscope was employed to characterize the morphologies of the gel networks. The operating voltage was 120 kV. For the measurements, all the gel samples were destroyed into flow state and 3 μL of the diluted sol solutions were carefully deposited on a Quantifoil R 1.2/1.3 100 Holey carbon films coated Cu 200 mesh grid. After blotting, the grid was rapidly inserted into liquid ethane. The frozen-hydrated samples were always stored in liquid nitrogen before the observation. The cryo-TEM images were recorded under low-dose conditions on a slow scan CCD camera (Gatan, model 830).

Dynamic light scattering (DLS) measurement

The formation of aggregates of **H** during aging was monitored using a Malvern Nano ZS 3600 Zetasizer. For the measurements, the solution of aged **H** was diluted to 4 mM by phosphate buffer (0.1 M, pH 7.0). The laser wavelength was 633 nm with a scattering angle of 173°. All the samples were allowed to equilibrate for 2 min and measured at 25 ^{o}C.

Molecular simulation and supporting discussion

To give additional insights into the aging of the hydrazide solution, we have performed coarse-grained Molecular Dynamic using the MARTINI force-field, which has been often applied to systems on the spatial and temporal scale usually not reachable by other techniques.[2] We simulated hydrazide (**H**) molecules solvated in water with a concentration of ~40 mM for 1.9 microseconds. Details of coarse-grained parameterization of **H** and simulations are similar to one done by Bochicchio et al.,[3] and are further present in ESI. At the early stages of the simulation the molecules assemble into small ordered stacks. The stacks interact with each other by merging into longer stack or by interacting with each other by the sides, resulting in small bundles. We observed that long singular stacks fragment into smaller fragments, while the stacks which form bundles do not fragment during the course of simulation.

Bundles of stacks seem to be more stable, probably due to a so called macrodipole interaction, i.e. interaction of a dipole created by many molecules stacked together. Macrodipole interaction for derivatives of trisamidecyclohexane (which is similar to hydrazide **H**) was recently studied in detail by Pereira Oliveira et al.,[4] where they show significant importance of the interaction when the stacks are close to each other. Since in the hydrazide case the centers of stacks in bundles are close to each other, we suspect that this is an important mechanism in the creation of stable small bundles, which can act as seeds for gelation.

Parameterization of hydrazide molecule: Hydrazide **H** was parameterized using the standard method described on cgmartni.nl. Similar parameterization has been done by

Bochicchio et al. in order to simulate self-assembly of 1,3,5-benzene-tricarboxamide (BTA).[3] We have parameterized a coarse-grained model to reproduce the dimerization free energy of an all atomistic model. All simulations were performed using GROMACS version 5.1.2.[5]

The all-atomistic parameterization of hydrazide **H** in the GROMOS 54A7 force-field was made using the Automatic Topology Builder.[6] The system was energy minimized with the steepest algorithm. Then the system was equilibrated consecutively in NVT ensemble and NPT ensemble. In all simulations the systems were coupled to a Berendsen thermostat with coupling constant 1.0 ps and a Berendsen barostat[7] with coupling constant 0.1 ps. For integration equations of motion a time step of 2 fs was used. Free energy has been calculated using umbrella sampling and Weighted Histogram Analysis Method (WHAM)[8] against the distance of the center of mass of two molecules. Results are present in Figure 1b (blue line).

The coarse-grained representation of **H** is present in Figure 1a. Three C1 beads were used to represent cyclohexane ring. Hydrazide groups were represented by a polar bead similar to a bead representing water in Polar MARTINI.[9] The hydrazide group is represented by three beads: one central ball which Lennard-Jones interactions with other balls and no charge, and two beads without Lennard-Jones interactions and charges +0.56e and -0.56e, respectively. The charges are kept at 0.14 nm distance from the central bead by distance constrains, and the angle between the negatively charged bead – central bead – positively charged bead was kept 180°, resulting in an effective distance between the charges of 0.28 nm. Such distribution of charges mimic dipole moment and allows to reproduce hydrogen bond interaction in a coarse-grained force-field. The system in coarse-grained representation was minimized with the steepest algorithm and then equilibrated in NVT and NPT ensemble. It was coupled to the v-rescale[10] thermostat with coupling constant 1 ps and Berendsen barostat with coupling constant 3 ps. For integration of equations of motion, we used 30 fs time step. Free energy of interaction of two molecules was calculated in the same way as for atomistic force field by umbrella sampling and WHAM analysis. The results are present in Figure 1b (orange line).

Free energy profiles are similar, and most importantly, reproduce exactly the level of interaction. The energy of dimerization for both all-atomistic and coarse-grained force-field, is approximate of -12 kJ/mol.

Finally, we have run 1.9 µs coarse-grained simulation of 21 molecules in water in 12 nm x 12 nm x 12 nm simulation box (resulting in concentration ~40 mM). During the course of the simulation small ordered stacks were formed, which can be seen as an initial rapid growth in number of hydrogen bonds (Figure 1d), and decrease of solvent accessible surface area (SASA) (Figure 1e). The small stacks interact with each other by forming longer stacks, or creating bundles of stack (and interacting by sides). Rapid decrease of number of hydrogen bonds and increase of SASA around 450 ns (Figure 1d-e) is a result of fragmentation of a large singular stack. It seems that stacks which form bundles consisting of two stacks are more stable.

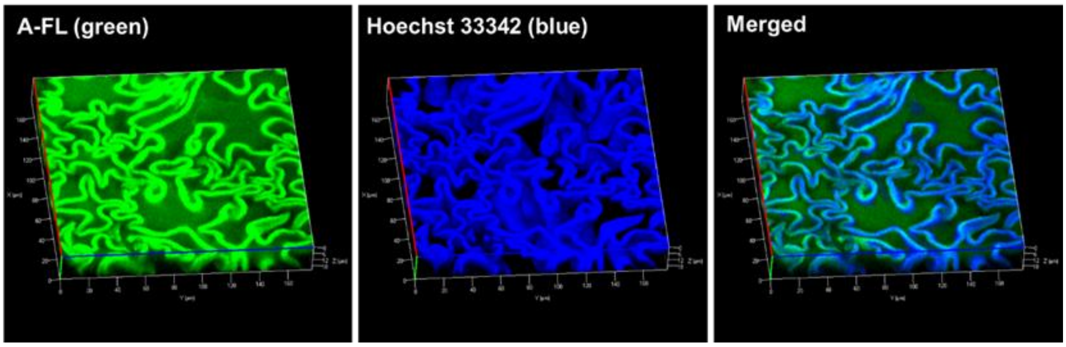

Figure S1. CLSM images of the heterogeneous hydrogels prepared with 20 mM fresh **H** and 120 mM (**A** + **A**⁻) (30 mol% **A**⁻).

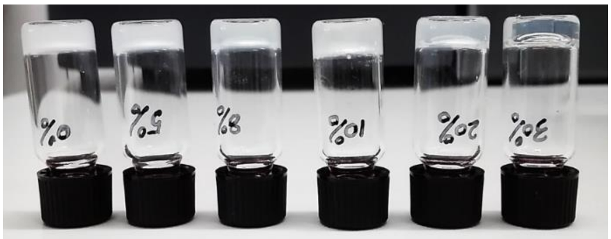

Figure S2. Photographs of gel samples prepared by 20 mM aged **H**, 120 mM (**A** + **A**⁻) (different mol% **A**⁻).

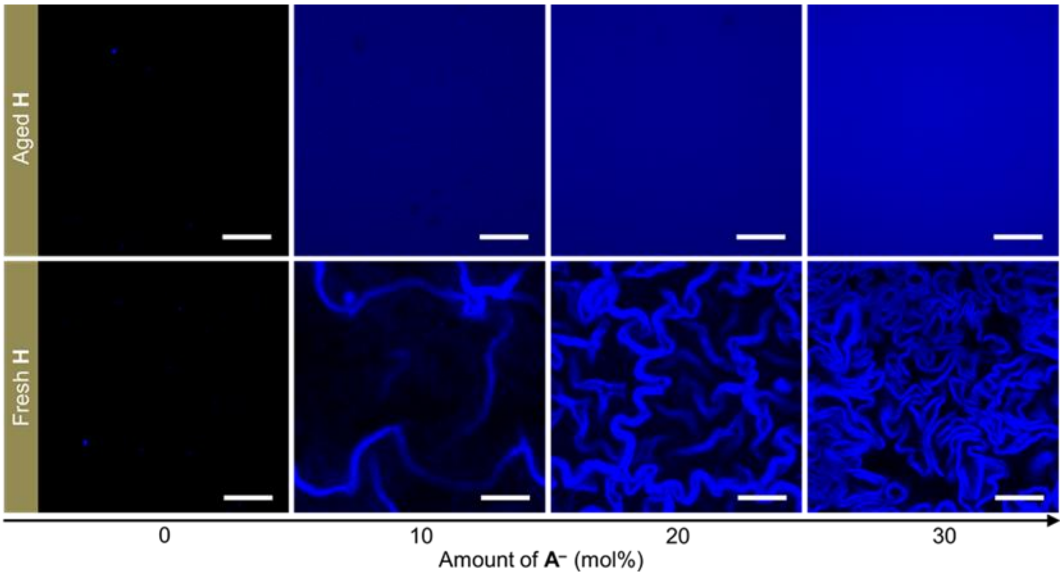

Figure S3. CLSM images (blue channel) of the morphologies of the gel networks prepared by aged (top) and fresh (down) **H** solutions as a function of the amount of **A**⁻, scale bars = 40 μm. Samples: [**H**] = 20 mM, [**A** + **A**⁻] = 120 mM (different mol% of **A**⁻), and [**Hoechst 33342**] = 30 μM.

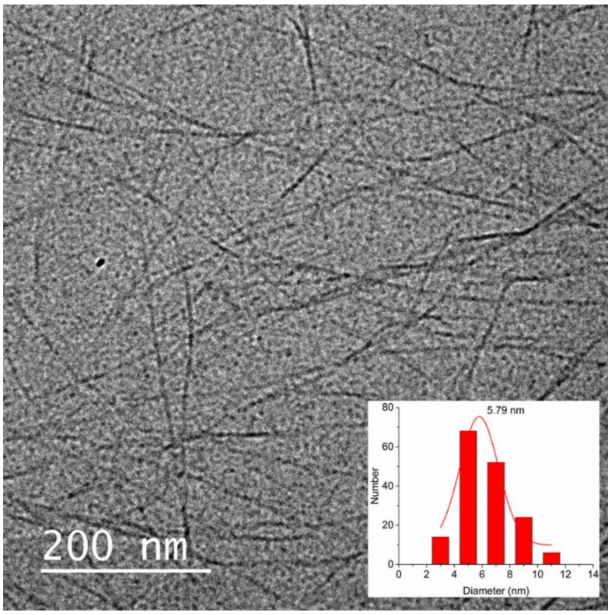

Figure S4. Cryo-TEM image and statistical diameter (inset) of the hydrazone fibers.

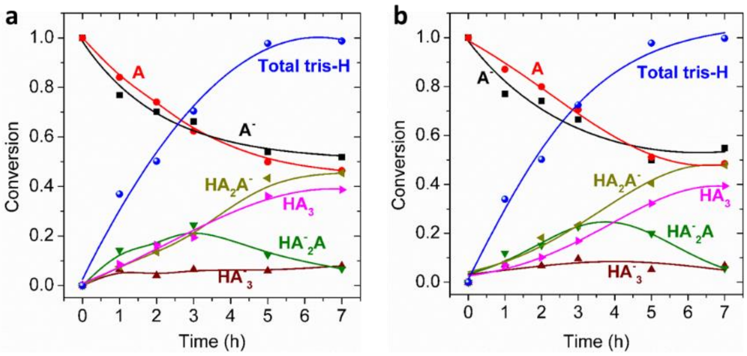

Figure S5. Formation of tris-hydrazone products and the consumption of aldehyde against time measured by HPLC: a) fresh **H**; and b) aged **H**. Samples: [**H**] = 20 mM, [**A** + **A**⁻] = 120 mM (30 mol% **A**⁻).

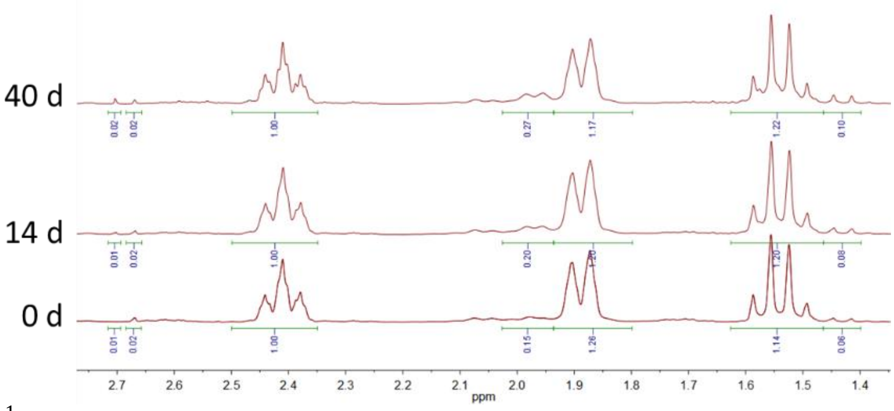

Figure S6. ^{1}H NMR spectra of **H** at different incubation time. The solvent for the measurement was D_2O. The impurities in the spectra during aging can be assigned to the very slight hydrolysis of **H** into the carboxyl types which has been tested to show no impact on the aging effects.

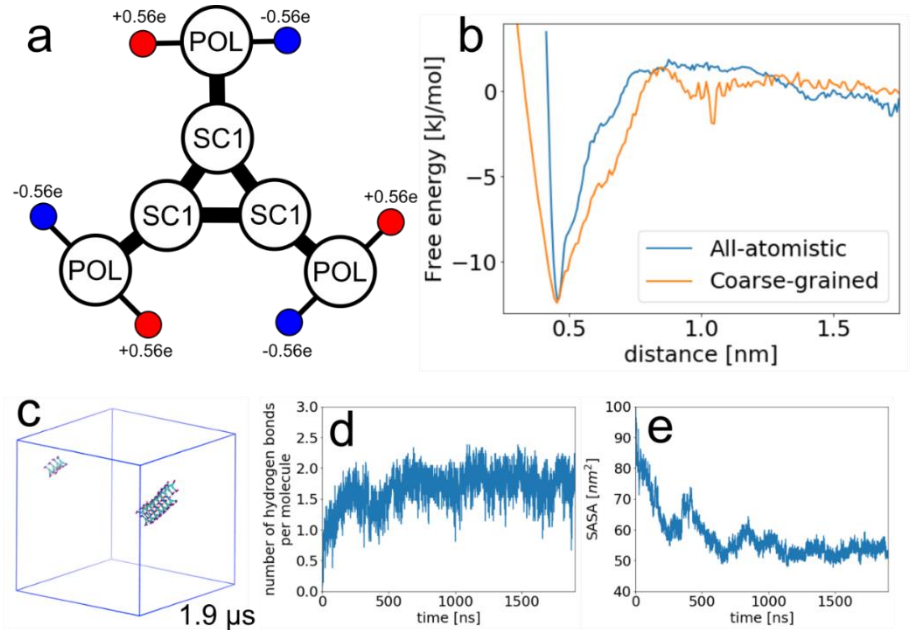

Figure S7. Coarse-grained simulations of hydrazide molecules. (a) Coarse-grained representation of hydrazide molecule. (b) Free energy profile of dimerization obtained by umbrella sampling of the distance between two molecules. (c) Snapshot of the final frame of the 1.9 μs coarse-grained simulation. The final structure consists of two stacks of molecules interacting by their side. (d) Progression of the number of hydrogen bond per molecule during the simulation. (c) Progression of solvent accessible surface area (SASA) during the simulation. At the beginning of simulation long singular stack was formed, which around 450 ns has fragmented into two smaller parts. After a while formed a cluster of two stacks seemed to have higher stability.

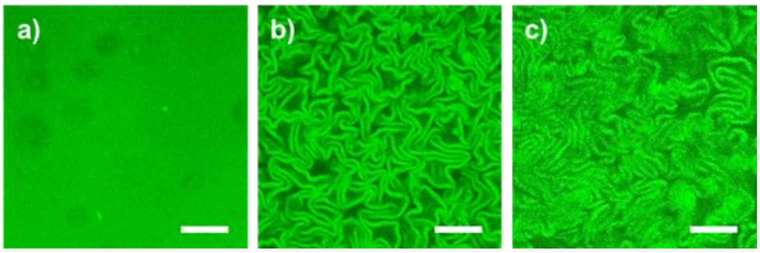

Figure S8. CLSM images of the gel samples prepared with a) **H** solutions aged in argon atmosphere; b) **H** solutions prepared from the **H** molecules obtained by freeze-dring and re-dissolving **H** seeds; and c) aged **H** solutions treated with THF. It can be seen from a) that aging **H** solution under the protection of argon still leads to formation of homogeneous gels, thus indicating the absent effects of CO_2 on the aging effects. Scale bars = 40 μm.

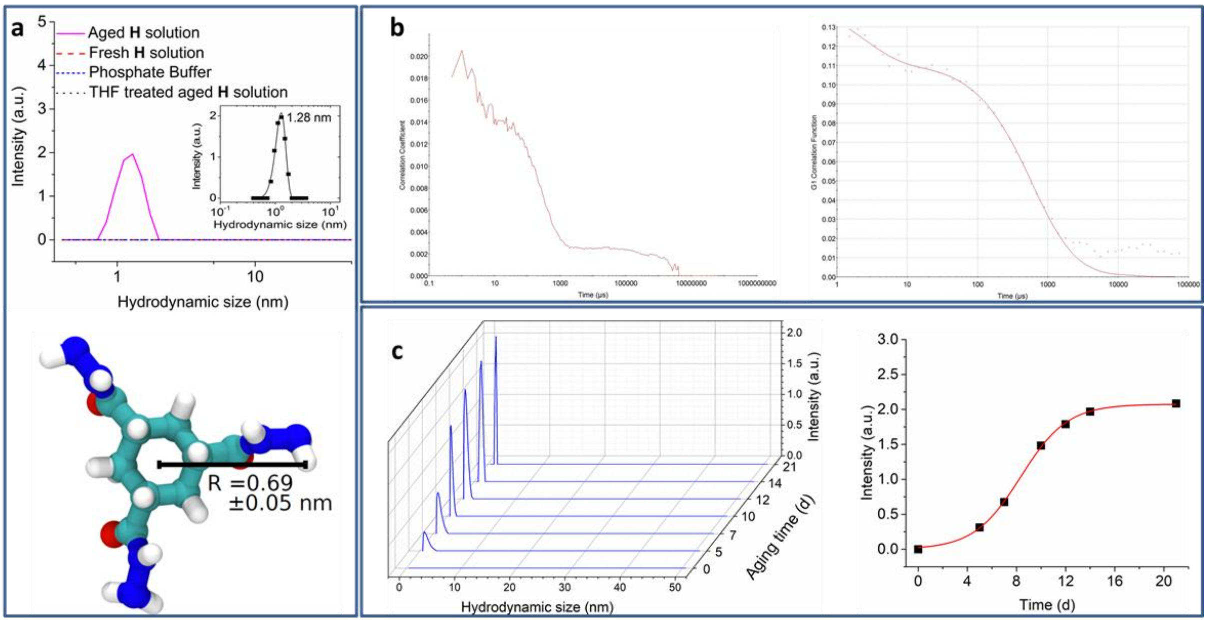

Figure S9. a) DLS measurements of fresh, aged, THF treated aged **H** solutions and blank phosphate buffer, and the theoretical size of a single **H** molecule determined by MD simulation. The theoretical size of **H** is slightly larger than the value that measured by DLS. But it is noteworthy that DLS obtains the size information by measuring the diffusion coefficient of the objects.[11] Therefore, the seed size of 1.28 nm measured by DLS means the nanoaggregate of H that have the same diffusion coefficient as a spherical **H** nanoparticle with a hydrodynamic diameter of 1.28 nm. Although the theoretical size of **H** molecule is slightly larger than the seed size measured by DLS, if we transform a single **H** molecule into a spherical **H** nanoparticle that has the same diffusion coefficient, its diameter should be, at least, smaller than 1.28 nm, that is why we cannot detect the scattering signal in the initial **H** solution using DLS; b) the correlogram and fitting curves, the imperfect fitting can be ascribed to the too small size of the seeds; and c) DLS measurements monitoring the aging process against time; the level off of the scattering intensity after two weeks indicating the termination of the seed growth which can be regarded as a dynamic equilibrium state where the rate of the self-assembly of **H** molecules into the seeds is equal to that of the disassembly of **H** molecules from the seeds. The concentration of **H** used for the measureuments is 4 mM, and the ages of aged H solutions are two weeks. Inset indicates the fitting curve of the scattering peak.

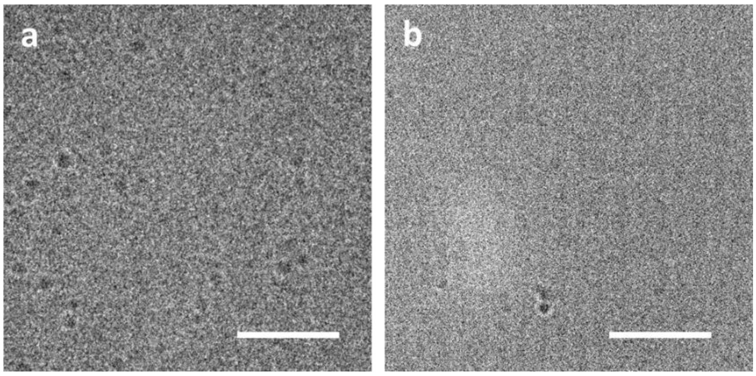

Figure S10. Cryo-TEM images of a) two weeks aged and b) freshly prepared **H** solution. Scale bars = 200 nm. The dark dots on these images are contaminations of ice crystals.

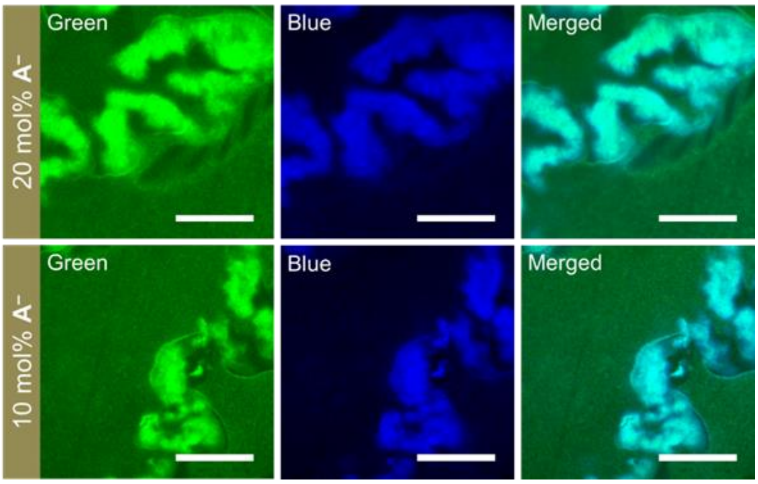

Figure S11. CLSM images of the homogeneous gel networks after three weeks incubation. Samples are prepared by 20 mM aged **H**, 120 mM (**A** + **A**$^{-}$) (different mol% **A**$^{-}$), 30 μM **A-FL** (green) and 20 μM **Hoechst 33342** (blue). Scale bars = 20 μm.

References

[1] a) J. Boekhoven, J. M. Poolman, C. Maity, F. Li, L. van der Mee, C. B. Minkenberg, E. Mendes, J. H. van Esch, R. Eelkema, *Nat. Chem.* **2013**, *5*, 433; b) J. M. Poolman, J. Boekhoven, A. Besselink, A. G. Olive, J. H. van Esch, R. Eelkema, *Nat. Protoc.* **2014**, *9*, 977; c) Y. Wang, M. Lovrak, Q. Liu, C. Maity, V. A. A. le Sage, X. Guo, R. Eelkema, J. H. van Esch, *J. Am. Chem. Soc.* **2019**, *141*, 2847.

[2] a) S. J. Marrink, H. J. Risselada, S. Yefimov, D. P. Tieleman, A. H. de Vries, *J. Phys. Chem. B* **2007**, *111*, 7812; b) P. W. J. M. Frederix, I. Patmanidis, S. J. Marrink, *Chem. Soc. Rev.* **2018**, *47*, 3470; c) D. Bochicchio, G. M. Pavan, *Adv. Phys. X* **2018**, *3*, 1436408.

[3] D. Bochicchio, G. M. Pavan, *Acs Nano* **2017**, *11*, 1000.

[4] M. P. Oliveira, H. W. Schmidt, R. Q. Albuquerque, *Chem. Eur. J.* **2018**, *24*, 2609.

[5] D. Van der Spoel, E. Lindahl, B. Hess, G. Groenhof, A. E. Mark, H. J. C. Berendsen, *J. Comput. Chem.* **2005**, *26*, 1701.

[6] A. K. Malde, L. Zuo, M. Breeze, M. Stroet, D. Poger, P. C. Nair, C. Oostenbrink, A. E. Mark, *J. Chem. Theory Comput.* **2011**, *7*, 4026.

[7] H. J. Berendsen, J. v. Postma, W. F. van Gunsteren, A. DiNola, J. Haak, *J. Chem. phys.* **1984**, *81*, 3684.

[8] S. Kumar, J. M. Rosenberg, D. Bouzida, R. H. Swendsen, P. A. Kollman, *J. Comput. Chem.* **1992**, *13*, 1011.

[9] a) S. O. Yesylevskyy, L. V. Schafer, D. Sengupta, S. J. Marrink, *Plos Comput. Biol.* **2010**, *6*, 1000810; b) D. H. de Jong, G. Singh, W. F. D. Bennett, C. Arnarez, T. A. Wassenaar, L. V. Schafer, X. Periole, D. P. Tieleman, S. J. Marrink, *J. Chem. Theory Comput.* **2013**, *9*, 687.

[10] G. Bussi, D. Donadio, M. Parrinello, *J. Chem. Phys.* **2007**, *126*, 014101.

[11] B. J. Berne, R. Pecora, *Dynamic light scattering: with applications to chemistry, biology, and physics*, Wiley, New York **1976**.
